# Successful Management of Septic Splenitis in an Abyssinian Cat

**DOI:** 10.1002/vms3.70943

**Published:** 2026-04-10

**Authors:** Martina Vecín Sancho, Perrine Henry, Mica Taylor, Catherine Davidson, María Lázaro Muñoz, Efa Llewellyn, Maša Vilfan, Paola Cazzini, Linda Morrison, Kelly Blacklock, Danièlle Gunn‐Moore

**Affiliations:** ^1^ The Royal (Dick) School of Veterinary Studies and the Roslin Institute The University of Edinburgh Midlothian UK

**Keywords:** pyogranulomatous splenitis, septic splenitis, splenitis, *Staphylococcus pseudintermedius*

## Abstract

A 3.5‐year‐old female neutered Abyssinian cat was referred for investigation of a 2‐week history of lethargy and intermittent vomiting, with recent development of diarrhoea, hyporexia, pyrexia, abdominal pain, moderate anaemia, hyperglobulinaemia and a palpably enlarged spleen. Abdominal ultrasound revealed a nodular spleen, and cytology identified septic splenitis (cocci were seen within neutrophils following fine needle aspiration). After 72 h of intravenous amoxicillin and clavulanic acid (20 mg/kg, per os, every 8 h), lack of clinical improvement prompted exploratory laparotomy and splenectomy. Histopathology was compatible with a suppurative to pyogranulomatous splenitis and identified intralesional bacteria. Bacterial culture of abdominal effusion and splenic biopsies grew *Staphylococcus pseudintermedius* resistant to benzylpenicillin. After surgery, the cat was treated with a 4‐week course of the fluoroquinolone pradofloxacin (5 mg/kg, per os, every 24 h) and made a complete recovery.

Septic splenitis is an uncommon diagnosis in the veterinary literature, being limited to eight splenic abscesses in cats, rare reports in dogs and a case of splenic foreign body in a cat and a heifer. To the authors’ knowledge, this is the first description of the successful management of septic splenitis in a cat not secondary to a foreign body. This case highlights that splenectomy should be considered the treatment of choice in the absence of response to antibiotic treatment and that antibiotic choice should be guided by antibiotic susceptibility results.

## Graphical abstract

A 3.5‐year‐old female neutered Abyssinian with pyrexia of unknown origin and splenomegaly was diagnosed with septic splenitis. Complete resolution of clinical signs followed splenectomy and antibiotic treatment with pradofloxacin. Septic splenitis, although uncommon, should be considered as a differential diagnosis in patients with splenomegaly and suspected immunosuppression or underlying infectious diseases. Splenectomy should be considered timely, particularly in the absence of clinical response to antibiotics.

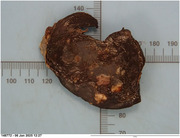

## 1 Case Description

A 3.5‐year‐old, female neutered Abyssinian cat weighing 2.95 kg was referred for investigations of a 2‐week history of lethargy and intermittent vomiting and a 1‐week history of diarrhoea and hyporexia. The cat had recently developed pyrexia and abdominal pain with a palpably enlarged spleen. A moderate non‐regenerative anaemia and hyperglobulinaemia were present on bloodwork.

The cat had unsupervised outdoor access, was up to date with vaccinations (including feline leukaemia virus) and received regular anthelminthic treatment for endoparasites and ectoparasites. Previous relevant medical history included a diagnosis of feline atopic syndrome (based on clinical signs) and chronic enteropathy (based on the presence of lymphoplasmacytic duodenitis on endoscopic biopsies) 2 years before presentation, a cat bite abscess 18‐months earlier and a suspected ascending cholangitis one year before presentation. The chronic enteropathy and atopic dermatitis were initially managed with a tapering course of prednisolone [Prednisolone, Millpledge Veterinary, Retford, UK, initial dose of 1.5 mg/kg every 24 h (q24h) per os (PO) being gradually decreased then stopped over the following 5 months], followed by tapering doses of ciclosporin (Atopica, Elanco, Hook, UK, initial dose of 7 mg/kg q24h PO, being gradually decreased and stopped over the following 7 months), alongside cyanocobalamin 0.5 mg q48h, PO for 12 weeks (Cobalaplex, Protexin Veterinary, Lopen Head, UK), a veterinary specific hydrolysed diet (Purina HA, Purina, York, UK) and occasional courses of topical antibiotics (fusidic acid), and corticosteroids (hydrocortisone aceponate). The cat bite abscess was managed with 7 days of amoxicillin and clavulanic acid 20 mg/kg q12h PO (Kesium, Ceva Animal Health Ltd, Wooburn Green, UK). The ascending cholangitis was treated with 4 weeks of amoxicillin and clavulanic acid 20 mg/kg q8h PO alongside supportive medications [maropitant 2 mg/kg q24h PO (Cerenia, Zoetis, Leatherhead, UK), mirtazapine 2 mg q24h transdermal (Mirataz, Dechra, Veterinary Products, Northwich, UK) and gabapentin 50 mg q12h PO for 5 to 7 days (Gabapentin, Summit Veterinary Pharmaceuticals, Kidlington, UK)]. Upon investigations of the latter, splenic changes were found on abdominal ultrasound, characterised by the presence of diffuse hypoechoic, often coalescing, nodules throughout the splenic parenchyma. The liver was largely normal with mild changes to the biliary tree (gall bladder sludge and tortuous cystic duct). During investigations for chronic enteropathy and cholangitis, the cat had repeatedly tested negative for *Toxoplasma gondii* (serum IgG and IgM) and feline retroviruses (Combo FIV/FeLV SNAP, IDEXX, Wetherby, UK). Three months before referral, a recrudescence of pruritus required a 3‐week tapering course of prednisolone with an initial dose of 2.5 mg/kg q24h PO for 7 days, decreased to 2.5 mg/kg q48h for a week, and 0.8 mg/kg q48h for 7 days, then stopped (Prednisolone, Millpledge Veterinary).

Physical examination on initial presentation to the referring veterinary surgeon revealed borderline tachycardia (200 beats per minute) and tachypnoea (50 breaths per minute) presumed secondary to stress. Cardiac and thoracic auscultation were unremarkable. The main clinical finding was moderate abdominal discomfort. The cat was initially treated for a presumptive recurrence of chronic enteropathy with injections of subcutaneous (SC) maropitant 1 mg/kg (Prevomax, Dechra Veterinary Products), intramuscular (IM) buprenorphine 0.02 mg/kg (Buprecare, Animalcare) and intravenous (IV) dexamethasone 0.1 mg/kg (Rapidexon, Dechra Veterinary Products), which transiently improved her demeanour and resolved the vomiting. The cat presented again 3 days later with pyrexia (39.7°C per rectum), marked abdominal pain and palpably abnormal spleen. Routine haematology performed at the time revealed a moderate non‐regenerative normocytic anaemia [haematocrit 17.9%, reference interval (RI): 30.3–52.3] and moderate leucocytosis characterised by moderate eosinophilia and mild neutrophilia, monocytosis and basophilia (Table 1). Serum biochemistry showed a mild increase in alanine aminotransferase (ALT 138 U/L, RI: 12–130) and moderate hyperglobulinaemia (73 g/L, RI: 28–51) (Table 1). Abdominal ultrasound was suspicious for splenomegaly, and multifocal hypoechoic splenic nodules were visible. A fine needle aspirate (FNA) cytology sample was obtained. Considering the complex presentation, the owner and referring veterinarian sought emergency referral to the Veterinary Teaching Hospital, and the cytological slides were handed to the local pathology laboratory on arrival.

**TABLE 1:** Haematology and serum biochemistry over the course of the disease.

	Initial presentation at the HfSA (31/12/2024)	First revisit (14/01/2025)	Second revisit (31/01/2025)	Reference interval
HCT (%)	*17.90	*24	*26	30.3–52.3
MCHC (g/dL)	36.90	32.80	32.8	28.1–35.8
MCV (fL)	36.40	42.8	42.5	35.9–53.1
RETIC (×10^9^/L)	11.80	54.1	47.6	3.0–50.0
WBC (×10^9^/L)	*24.45	10.72	11.01	2.87–17.02
NEU (×10^9^/L)	*14.30	5.68	4.40	2.3–10.29
LYM (×10^9^/L)	4.85	3.97	5.51	0.92–6.88
MONO (×10^9^/L)	*0.94	*0.75	0.33	0.05–0.67
BASO (×10^9^/L)	*1.48	0.0	0.0	0.01–0.26
EOS (×10^9^/L)	*2.88	0.97	0.77	0.17–1.57
PLT (×10^9^/L)	289	544	248	300–600
ALB (g/L)	26	25.6	29.6	22–40
GLOB (g/L)	*73	*54.6	39.3	28–51
ALB/GLOB	0.4	0.5	0.8	
TP (g/L)	*99	80.2	68.9	57–89
ALKP (U/L)	18	19	23	14–111
ALT (U/L)	*138	96	71	12–130
CREA (µmol/L)	43	78	97	71–212
UREA (mmol/L)	4.6	6.7	7.9	5.7–12.9
GLU (mmol/L)	8.29	−	5.5	4.11–8.84
Cl (mmol/L)	116	119	117	112–129
K (mmol/L)	4	4.6	4.2	3.5–5.8
Na (mmol/L)	158	149	149	150–165

*Note*: The results with an asterisk indicate an abnormal value.

Abbreviations: ALB, albumin; ALB/GLOB, albumin‐globulin ratio; ALKP, alkaline Phosphatase; ALT, alanine transaminase; BASO, basophils; Cl, chloride; CREA, creatinine; EOS, eosinophils; GLOB, globulin; GLU, glucose; HCT, haematocrit; K, potassium; LYM, lymphocytes; MCH, mean corpuscular haemoglobin; MCHC, Mean corpuscular haemoglobin concentration; MCV, mean corpuscular volume; MONO, monocytes; Na, sodium; NEU, neutrophils; PLT, platelets; Retic, reticulocytes; TP, total protein; WBC, white blood cells.

Upon referral, the cat was quiet but alert and responsive. Heart rate and respiratory rate were increased (208 beats per minute and 40 breaths per minute, respectively), with normal effort and auscultation. Pulses were synchronous but hyperdynamic. Mucous membranes were pink with a normal capillary refill time. Hydration status was adequate, and blood pressure was mildly low (Doppler, average systolic 116 mmHg) (Simpson et al. 2007, Payne et al. 2017). Abdominal palpation was uncomfortable and suggestive of splenomegaly. Body temperature was mildly elevated (38.9°C axillary). Thoracic and abdominal point‐of‐care ultrasound were negative for effusion using standardised techniques, and the left atrium to aorta ratio was within normal limits (Lisciandro 2021). Manual packed cell volume (PCV) was 24% (RI: 25–45), and total solids were markedly increased (104 g/dL, RI: 60–75) in keeping with the previously documented moderate hyperglobulinaemia. The bedside assay for feline viruses (Combo FIV/FeLV SNAP) was negative.

Differential diagnosis for splenomegaly included benign causes of congestion (sedation, splenic vein thrombosis, splenic torsion, portal hypertension, and right sided heart failure), reactive changes (extramedullary haematopoiesis, nodular hyperplasia, and haematoma), infiltrative disease (hypereosinophilic syndrome), neoplasia (mast cell tumour, multiple myeloma, lymphoma and haemangiosarcoma), infectious diseases (feline infectious peritonitis (FIP), toxoplasmosis, bartonellosis, haemotropic mycoplasmosis, ehrlichiosis, cytauxzoonosis and histoplasmosis) and granulomatous splenitis (tuberculosis, non‐tuberculous mycobacteriosis) (Spangler and Culbertson 1992; Côté et al. 2024). In this case, the most likely differential diagnoses were neoplastic or infectious disease since hyperglobulinaemia is commonly seen with both paraneoplastic syndrome or antigenic stimulation. Sedation‐related congestion was excluded by the history, and heart failure was less likely given the absence of cardiac changes on point‐of‐care ultrasound. Trauma was not reported, making haematoma unlikely. The hyperdynamic pulses were presumed secondary to the moderate anaemia, as the cat was otherwise euvolaemic and euhydrated with no overt signs suggestive of septic shock. The anaemia was suspected to be secondary to chronic inflammatory disease, although other causes such as haemophagocytic syndrome, bone marrow disease, pre‐regenerative anaemia, or sequestration were also possible.

**FIGURE 1:**
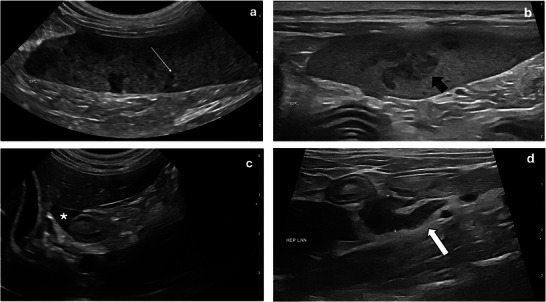
(a) Microconvex transducer and (b) linear transducer ultrasonographic still images of the body of the spleen. The thin white arrow points to an example of the many small, hypoechoic, discrete nodules noted throughout the spleen. The thick black arrow points to an example of the larger, hypoechoic coalescing nodules. (c) Microconvex transducer and (d) linear transduced ultrasonographic still images of the peritoneum. The white asterisk is within a pocket of free peritoneal fluid, just caudal to the liver. The white callipers labelled D1 measure the short axis of the abnormal hepatic lymph node described. The thick white arrow points to the mild perinodal steatitis.

**FIGURE 2:**
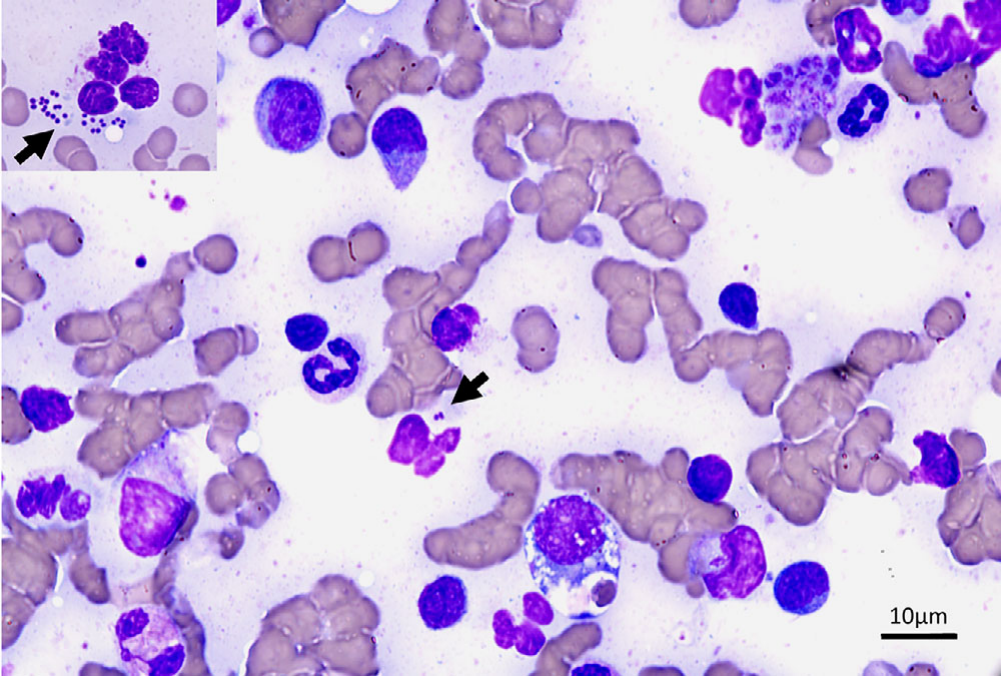
Splenic fine needle aspirate. A few variably preserved and often degenerate neutrophils are seen, and they occasionally contain cocci bacteria (black arrow). A heterogeneous population of lymphocytes, a few eosinophils and macrophages with occasional erythrophagocytosis are also seen on a light proteinaceous background with moderate blood contamination. Modified Giemsa stain, 1000X magnification. Scale bar 10 µm. Inset: cocci bacteria (black arrow).

While waiting for the cytology report on the splenic aspirates acquired by the referring veterinarian, further investigations were conducted. Thoracic radiographs were performed to screen for the presence of metastasis and failed to reveal significant thoracic pathology. Abdominal ultrasound confirmed the suspected splenic changes. The spleen was enlarged (increased thickness up to 13.8 mm; RI: 8.2 ± 1.4 mm) (Reese et al. 2013), with diffuse, irregularly marginated, ill‐defined, coalescing, hypoechoic nodules measuring up to 1 cm in width, giving it an overall mottled, ‘honeycomb’ appearance (Figure 1a,b). Additionally, there was moderate peri‐splenic steatitis and locoregional lymphadenopathy. The hepatic lymph nodes were the most affected, being rounded, hypoechoic and moderately enlarged at 9 mm in short axis. A moderate volume of hypoechoic, mildly flocculant peritoneal fluid was also present diffusely, being most marked between the body of the spleen and the left kidney (Figure 1c,d). Ultrasound‐guided FNA samples of the spleen were repeated as results from the previous samples were not yet available, and their diagnostic quality was unknown. Abdominocentesis and cystocentesis were also performed, without immediate complication. In‐house examination of the abdominal effusion revealed neutrophilic inflammation, seemingly sterile based on the absence of visible bacteria; however, the sample was submitted for bacterial culture. Protein content and cellularity were not formally assessed. Urinalysis was unremarkable. Cytologic examination of the splenic aspirates identified a septic neutrophilic inflammation characterised by variable degenerate neutrophils, rarely containing bacteria, rare extracellular aggregates of cocci bacteria, some extramedullary haematopoiesis and marked stromal cell and lymphoid reactivity (Figure 2). Cytology acquired during referral investigations returned similar findings, albeit without neutrophilic degeneration or obvious microorganisms, despite the absence of interference from antibiotic treatments.

The cat was treated for septic splenitis with IV fluid therapy at 2 mL/kg/h (Lactated Ringer's solution, Aqupharm, Ecuphar), empiric broad spectrum antibiotic therapy with amoxicillin‐clavulanic acid 20 mg/kg q8h IV (Co‐amoxiclav, Sandoz, Switzerland), antiemetic with maropitant 1 mg/kg q24h IV (Vetemex, Virbac, Carros, France) and analgesia with gabapentin 50 mg q8h PO (Gabapentin, Summit Veterinary Pharmaceuticals) and buprenorphine 0.02 mg/kg q8h IV (Buprelieve, Zoetis).

**FIGURE 3:**
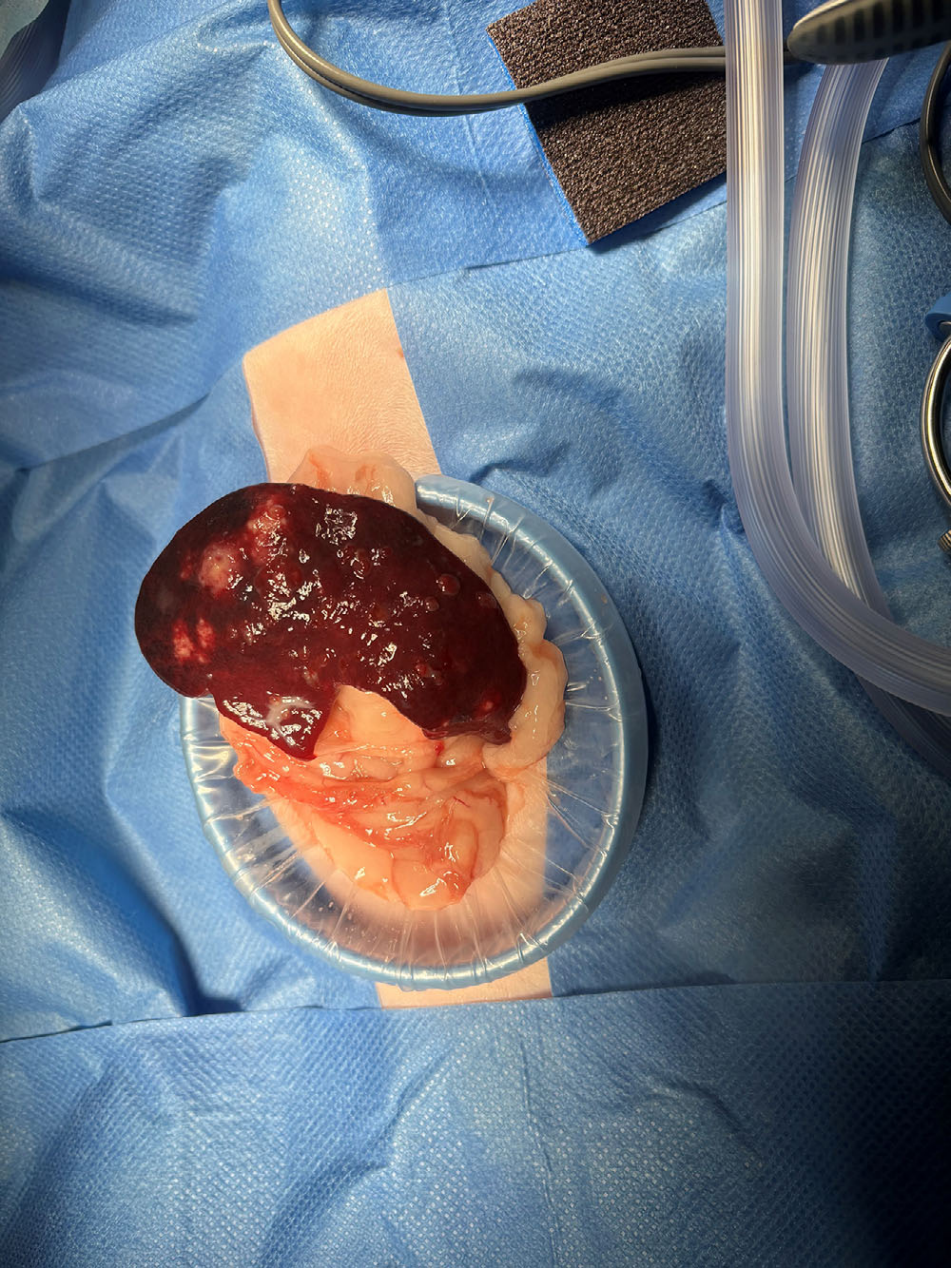
Intraoperative image of the spleen. Note the dark colouration and the multifocal yellow‐brown regions.

After 72 h of medical management, the cat's clinical condition remained static (ongoing hyporexia, pyrexia and unchanged PCV), which prompted splenectomy. Under general anaesthesia, the exteriorised spleen was markedly abnormal, with a dark purple‐brown colour and multifocal to coalescing pale yellow‐brown regions (Figure 3). Splenectomy was performed by cauterising hilar vessels using a tissue sealer (ENSEAL X1 Large Jaw Tissue Sealer, Ethicon Inc., Birkenhead, UK). No other gross abnormalities were identified in the vicinity of the spleen; samples were submitted for microbiology and histopathology. An oesophageal feeding tube was placed under general anaesthesia to facilitate post‐operative enteral nutrition and drug administration.

Recovery was uneventful. Analgesia was provided with methadone IV 0.2 mg/kg q4h (Comfortan, Dechra Veterinary Products), then de‐escalated to buprenorphine 0.02 mg/kg q8h IV the day after surgery. Gradual enteral nutrition was provided through the feeding tube until the appetite returned, 5 days after placement (33% of the resting energy requirements were given on day one, increasing to 66% on day 2 and 100% on day 3). Over the post‐operative hospitalisation period (3 days), the cat's body weight increased (to 3.07 kg on discharge; a 4% increase), as did her PCV (26%; was 24%), and total solids decreased (94 g/L; was 104 g/L). The cat started eating 50%–60% of the food offered immediately post‐surgery and was discharged on oral antibiotics, amoxicillin and clavulanic acid 20 mg/kg q12h PO (Cladaxxa, Krka UK Ltd., Langley, UK).

**FIGURE 4:**
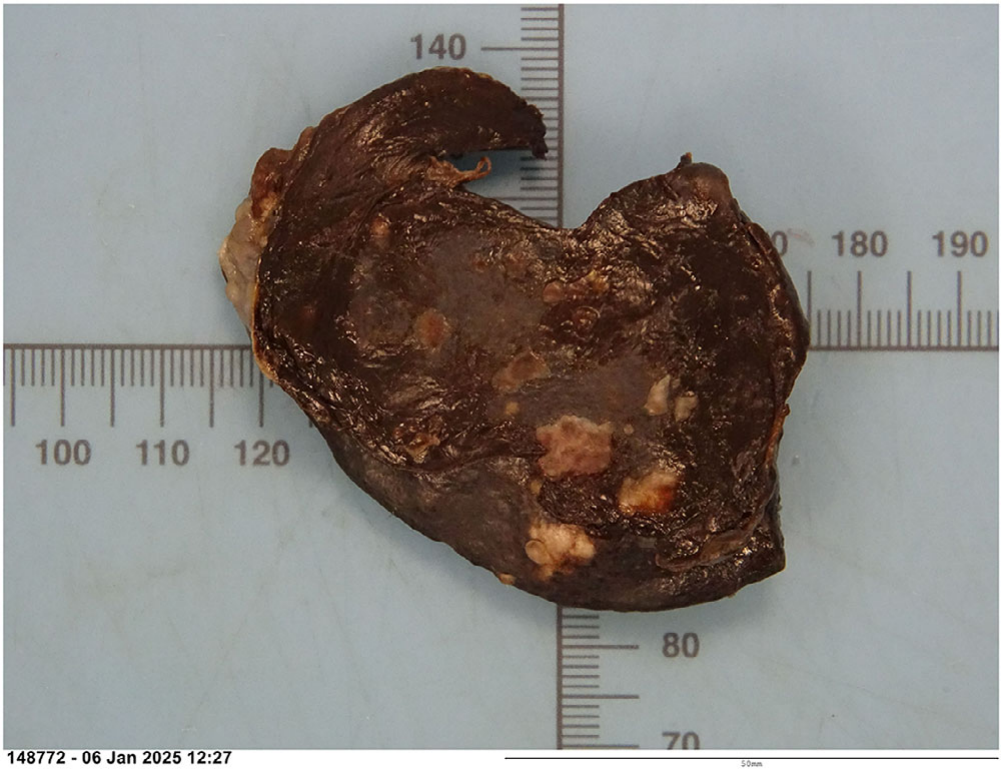
Gross features of the formalin fixed spleen. Spleen measuring 8×3–4.5×1.53 cm, dark purple, brown with multifocal to coalescing pale yellow brown regions. (Image courtesy of Dr Adrian Philbey). Scale bar: each graduation is 1 mm.

**FIGURE 5:**
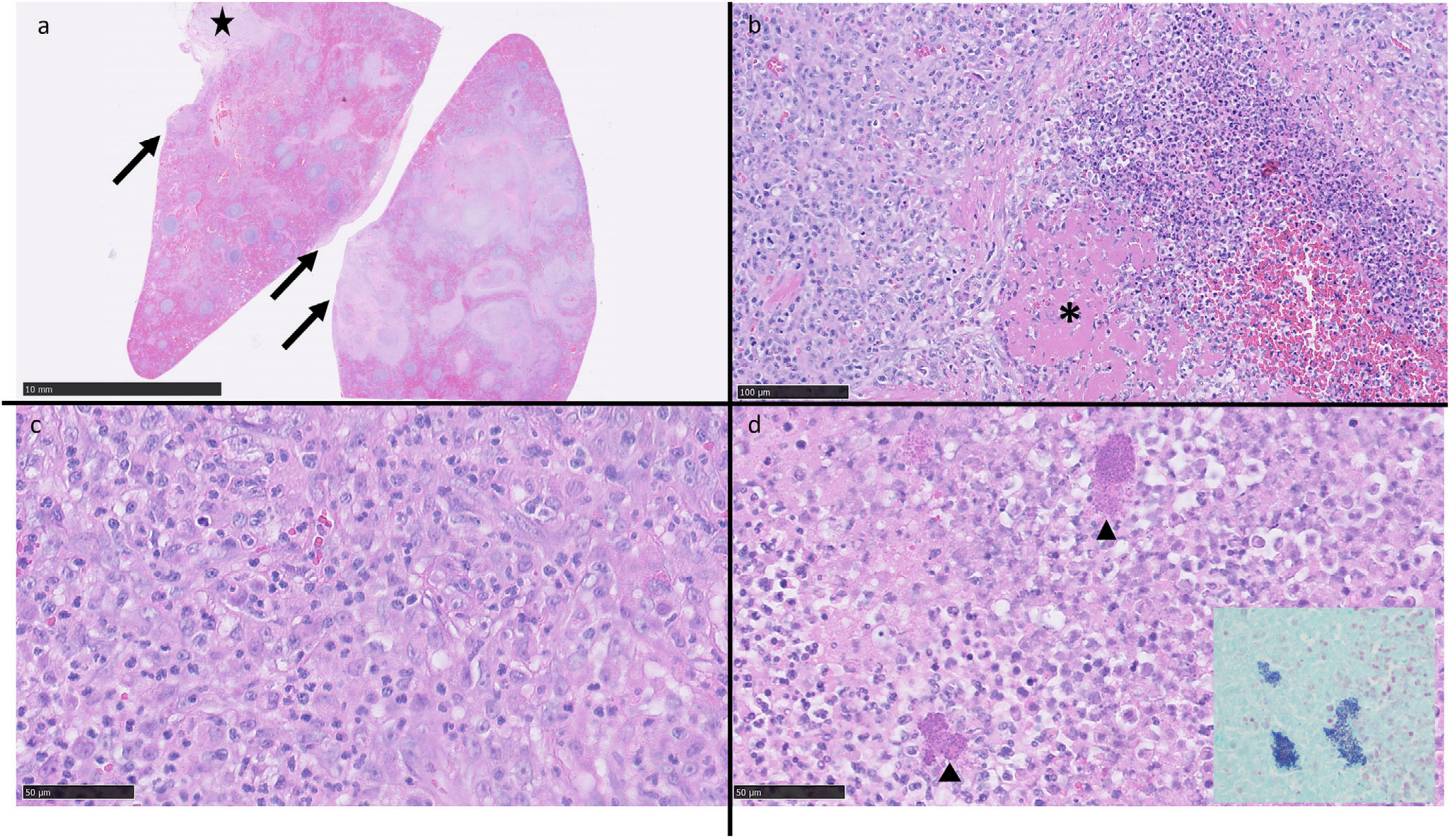
Splenic histopathology. (a) Multifocal to coalescing areas of inflammation throughout the spleen extending into attached mesentery (star) and onto the capsular surface (arrows). H&E Scale bar = 10 mm. (b) Fibrinoid necrosis (asterisk) within area of inflammation. H&E 20X magnification Scale bar = 100 µm. (c) Pyogranulomatous inflammation. H&E 40X magnification, scale bar = 50 µm. (d) Suppurative inflammation with intralesional bacteria (arrowheads). Inset: Bacteria are Gram positive. H&E and Gram, 40X magnification, scale bar = 50 µm.

Splenic histopathology identified a severe, multifocal to coalescing, subacute, suppurative to pyogranulomatous and necrotising splenitis with intralesional bacterial colonies, capsulitis and peritonitis. Gram stain identified gram‐positive cocci (Figures 4 and 5). Two days after discharge, bacterial culture results from splenic samples obtained at the time of splenectomy and from the abdominal effusion returned a light growth of *Staphylococcus pseudintermedius* resistant to benzylpenicillin. Antibiotic therapy was adjusted based on these results to pradofloxacin 5 mg/kg q24h PO (Veraflox 25 mg/mL, Elanco) for 4 weeks. Clinical signs steadily improved following antibiotic change with a gradual return of spontaneous appetite.

Upon scheduled reassessment 7 days post‐discharge, repeat haematology showed a resolution of the previous changes (anaemia and leucocytosis), and biochemistry showed a marked improvement in blood proteins (globulins decreased from 73 g/L to 55 g/L; Table 1). The feeding tube was removed as the cat had been covering her full energy requirements by mouth for several consecutive days. Serology for *Bartonella henselae* was negative (titres <1:64). At the following recheck, one month post splenectomy, the cat remained clinically well, and all blood parameters had normalised (Table 1). Pradofloxacin was continued for another 2 weeks. The clinical parameters, medical and dietary management over the course of treatment, are summarised in Table S1. At the time of writing, 8 months after initial presentation, the cat is still reported to be clinically well.

Upon review of the case after discharge, immunohistochemistry for feline coronavirus antibodies as well as Ziehl‐Neelsen to identify acid‐fast bacteria were performed on splenic tissue, both yielding negative results. An ELISA rapid test for vector‐borne diseases (IDEXX SNAP 4Dx Plus Test) was also negative.

## 2 Discussion

To the authors’ knowledge, this case report is the first to describe the successful treatment of septic, suppurative to pyogranulomatous and necrotising splenitis in a cat not secondary to a foreign body. Septic splenitis is extremely uncommon in cats, with only nine cases reported in the literature. Of these, eight were reported by Spangler and Culbertson (1992) in a retrospective study describing the findings from 455 splenic histopathology samples from cats; the eight cases were characterised by infiltrates of neutrophils, often forming abscesses with intra‐lesional bacteria. The ninth feline case was a report of septic peritonitis secondary to a vegetal foreign body embedded in the spleen (Culp and Aronson 2008). Splenectomy was performed, and histopathology revealed acute fibrinous splenitis associated with a plant‐based foreign body, but no overt infectious agent. Successful management of septic splenitis not associated with foreign bodies has not, to the authors’ knowledge, been reported in cats.

In veterinary medicine, previous reports of septic and pyogranulomatous splenitis have mainly involved dogs and cattle, but a case report describes non‐infectious pyogranulomatous splenitis in a cat following splenic torsion (Bucknoff and Rolph 2024). In dogs, pyogranulomatous splenitis has been previously identified as associated with fungal, protozoan and bacterial infections, usually in the form of single case reports. Septic splenitis has been described as associated with splenic abscesses (Abdellatif et al. 2014) and disseminated mycobacteriosis and listeriosis (Schroeder and van Rensburg 1993; Campora et al. 2011), amongst others. Another study in dogs identified bacterial growth in 35% of surgically excised spleens irrespective of the disease affecting the organ. In that study, the most commonly identified bacteria included *Staphylococcus* spp., *Enterococcus* spp., and *Klebsiella pyogenes* (Richardson and Brown 1996). In another study, splenitis identified in dogs was deemed secondary to other diseases such as neoplasia, gastric dilation‐volvulus, and septic inflammation, causing bacterial translocation. Many cases had intra‐lesional bacteria, but some also had concurrent *Hepatozoon canis* meronts, *Leishmania infantum* amastigotes or fungi (Ferri et al. 2017). Another cause of mononuclear infiltration of the spleen is experimental infections with *Ehrlichia* spp. (de Castro et al. 2004).

In cattle, septic splenitis has been described mostly secondary to a traumatic foreign body (Rosenberger and Gründer 1970; Stöber 2002; Radostits et al. 2007). The disease is typically diagnosed during necropsy. Antibiotic treatment often results in a transient resolution of the pyrexia but is not curative. Surgical management has only been reported once, in a 30‐month‐old heifer, as suppurative splenitis is regularly complicated by concurrent peritonitis and marked fibrinous adhesions, entailing a poor prognosis (Nuss et al. 2009). In that report, septic splenitis was suspected based on imaging findings suggestive of abscesses and the presence of a penetrating reticular foreign body. Splenectomy was successful, and the heifer recovered uneventfully with peri‐operative administration of IV antibiotics (amoxicillin) but later developed septic arthritis and was ultimately euthanised. The septic arthritis was presumed secondary to impaired immune function in the absence of splenic immunological activity as reported in human medicine, although concurrent bacteraemia could not be excluded (Nuss et al. 2009; Luu et al. 2019).

In human medicine, septic splenitis and splenic abscesses remain rare, but they have been more extensively described, commonly in association with immunosuppression (e.g., human immunodeficiency virus or chemotherapy). Other predisposing factors have also been identified, including the presence of distant infection (e.g., bacterial endocarditis), splenic trauma, malignant neoplasia, haemoglobinopathies, pancreatic disease and diabetes mellitus (Choudhury et al. 2006; Shaparia et al. 2022). Septic splenitis and splenic abscesses are regularly suspected to be secondary to septic emboli and bacteraemia (Radcliffe et al. 2022). However, the relationship between sepsis and septic splenitis is contentious. Several studies question the link of causality between the identification of septic splenitis and the presence of sepsis (Wise 1976; Feig and Cina 2001), while others suggest that the lack of association could represent undocumented use of antibiotics before death (Arismendi‐Morillo et al. 2004). Regardless, splenectomy along with antimicrobial therapy has traditionally been the treatment of choice for splenic abscesses in humans, with success rates of 100% (Tung et al. 2006). Other studies have reported the successful use of antibiotic therapy alone or combined with ultrasonography‐ or computed tomography‐guided drainage to maintain the immunologic functions of the spleen (Choudhury et al. 2006; Radcliffe et al. 2022).

In the present case, bacterial culture of the abdominal effusion and splenic biopsies returned a light growth of *S. pseudintermedius*. This is an opportunistic pathogen belonging to the skin microbiota of healthy dogs and arguably in cats (Bierowiec et al. 2021). Its identification in abdominal fluid sampled transcutaneously under ultrasound guidance introduced the possibility of skin contamination. However, this was rendered less likely by the identification of the same bacteria on splenic tissue obtained surgically. Underlying immunosuppression could, potentially, have allowed small numbers of *S. pseudintermedius* introduced when initially aspirating the spleen to result in apparent *S. pseudintermedius*‐ splenitis, obscuring the primary reason for the cat becoming unwell. However, given the marked splenic changes and presence of abdominal effusion observed on ultrasound before the initial sampling, iatrogenic contamination of the spleen and abdominal cavity is considered unlikely. A pre‐existing pathological process is therefore favoured. In the absence of a history of penetrating trauma to the spleen, a localised bacterial infection is unlikely, and the most plausible scenario for this cat's splenitis remains transient bacteriaemia with *S. pseudintermedius* from a skin infection, with subsequent seeding to the spleen, secondary to immunosuppression.

Despite extensive investigations, the definitive cause for the splenitis was not identified; i.e., how did the cat's spleen become infected with *S. pseudintermedius*? Several hypotheses are possible, including chronic immunosuppression and/or undocumented bacteriemia. The cat had received multiple courses of immunosuppressive medications for the management of feline atopic syndrome and chronic enteropathy, which could have predisposed it to immune suppression. Given the history of feline atopic syndrome, with previous skin ulceration, this could easily have resulted in *S. pseudintermedius* bacteraemia, with these bacteria remaining sequestered in the spleen for 2 years. This cat was also the smallest and least vigorous kitten in the litter, raising the possibility of failure of passive immune transfer, with lifelong immunosuppression. The history of a cat bite abscess, one year before presentation, invited testing for *Bartonella henselae*. This was further supported by a human report of necrotising and granulomatous splenitis secondary to bartonellosis (Liston and Koehler 1996) and by a case report of *Enterococcus faecalis* bacteraemia facilitated by chronic immunosuppression from *B. henselae* endocarditis (Colella et al. 2023). Although *Bartonella* spp. serology was negative; bartonellosis could not be entirely excluded due to the low sensitivity of serological tests (Álvarez‐Fernández et al. 2022) and the possibility of an impaired immune response, which may have prevented the development of detectable antibodies. However, regardless of the modality, the diagnosis of bartonellosis remains difficult due to the overall poor sensitivity of the ancillary tests available (i.e., serology, PCR, and enrichment culture) and requires a careful interpretation of the patient's clinical signs, as positive results do not indicate disease causation (Álvarez‐Fernández et al. 2022). Further testing for bartonellosis would have been of value in the present case, particularly if undertaken before antibiotic therapy was implemented. By the time this theory was entertained, the cat had already received 14 days of antibiotics, which further justified not pursuing molecular identification or enrichment culture.

Extensive investigations into other possible causes of bacteraemia were also considered, but not undertaken for a number of reasons. Echocardiography and measurement of cardiac troponin I to rule out endocarditis were declined by the owner for financial reasons. Blood cultures were not pursued due to concurrent anaemia, making sampling of moderate amounts of blood undesirable, as well as concurrent antibiotic therapy, which would have negatively affected the test's sensitivity (Greiner et al. 2007). If these tests had returned positive results, the cat would have been diagnosed with infectious endocarditis (Sereda et al. 2022). The most common cause of infectious endocarditis in cats is *Bartonella* spp. although other infectious agents are possible, e.g., *S. pseudintermedius* in the current case. Antibiotic therapy should have been guided by antibiotic sensitivity testing derived from the blood cultures and implemented for a duration of 6 weeks.

Histopathology of the spleen revealed suppurative and pyogranulomatous inflammation. The most common infectious diseases associated with pyogranulomatous infiltrates in cats include FIP, toxoplasmosis and mycobacteriosis; however, it can also be associated with other chronic infections (O'Brien et al. 2021). Immunohistochemistry (IHC) for feline coronavirus antibodies and Ziehl Nielsen stains to identify acid‐fast bacteria were both negative. Splenic aspirates could also have been assayed by quantitative FCoV RT‐PCR and/or mycobacterial PCR; however, the negative results from the other assays, when combined with the other findings, meant these infections were unlikely. Previous serology for *T. gondii* had been repeatedly negative, and no protozoan oocysts were seen on histopathology, making toxoplasmosis unlikely. Nevertheless, histopathology is only moderately sensitive for detecting protozoan oocysts (Liu et al. 2015), so the authors cannot completely exclude recent toxoplasmosis infection from predation.

Other infections were also considered. The cat had no known exposure to ticks, and an in‐house ELISA test for tick‐borne pathogens was negative. Given the high sensitivity and specificity of this assay, and considering cost limitations*, Ehrlichia canis* serology was not submitted (Stillman et al. 2014). Specific PCR testing for haemotropic mycoplasmosis (i.e., *M. haemominutum, M. haemofelis, Ca. M. turicensis*) was not conducted. This was mainly driven by cost limitations and the low probability of these infections being the cause of the cat's clinical signs. For example, the cat presented a non‐regenerative anaemia rather than a strongly regenerative one typically seen with haemotropic mycoplasmosis. However, the anaemia associated with these infections can occasionally be non‐regenerative. *Cytauxzoon felis* and *Histoplasma capsulatum* testing was not conducted as the cat had no travel history outside of Scotland, where these diseases are not endemic, and no organisms were seen on cytology and histopathology. On balance, the diagnosis of *S. pseudintermedius* as the cause of the suppurative to pyogranulomatous inflammation identified in this cat and the positive response to treatment with pradofloxacin make most of these potential infections less likely. Regardless of the nature of the infection, underlying immunosuppression is likely to have played a role in the development of this cat's splenitis.

Splenic cytologic samples obtained at the referring practice revealed cocci bacteria, while samples obtained after referral only revealed neutrophilic inflammation. This is most likely due to the sampling of different areas within the parenchyma of the spleen (e.g. sampling variability), combined with the nonuniform distribution of bacteria and the highly focal nature of samples obtained via FNA. In a recent study comparing splenic ultrasound‐guided FNA with splenic excisional histopathology, the specificity of FNA was only 72%, and the sensitivity was 71% (Holter et al. 2023). Nevertheless, due to its minimally invasive nature, lower cost, and rapid turnaround time, cytology is still recommended as a first‐line diagnostic tool (Valenciano and Cowell 2020). Similarly, in‐house cytology of the peritoneal effusion revealed no bacteria, but bacterial culture of the fluid identified a light growth of *S. pseudintermedius*. This is a known limitation of cytological examination of samples in the diagnosis of septic effusions, which only has a moderate sensitivity (75%–83%) (but high specificity of 93%–100%) (Allen and Evans 2022; Medardo et al. 2024). The sensitivity could have been increased by submitting the sample for examination by a board‐certified veterinary clinical pathologist. Additionally, despite the absence of visible bacteria, if neutrophilic inflammation is observed, culture is always indicated to further exclude an infectious process.

In the absence of bacteria being seen on fluid cytology, the abdominal effusion was presumed to be sterile despite a high neutrophil count. Unfortunately, that sample was not characterised further (i.e., no cellular count or measure of protein content was undertaken), which limits its interpretation, and the fluid could represent either a reactive peritoneal effusion or, more likely, a neutrophilic exudate. In hindsight, it is plausible that this cat had septic peritonitis, the management of which would have been surgical (i.e., removal of the source of infection) (Anderson et al. 2021). The haemodynamic changes noted on admission (i.e., the borderline tachycardia, mildly low blood pressure and hyperdynamic pulses), along with the leucocytosis and pyrexia, raise the question of early (compensated) septic shock. Although this would not have fitted the sepsis criteria, it would be compatible with systemic inflammatory response syndrome (SIRS) (Cortellini et al. 2024). Regardless, the authors believe that this cat would have benefited from earlier emergency abdominal surgery.

Treatment initially relied on broad‐spectrum antibiotics (i.e., amoxicillin and clavulanic acid); splenectomy was only considered when the cat failed to improve. A mild improvement of the cat's demeanour and appetite was observed two days after surgery, whilst she was still being treated with amoxicillin and clavulanic acid. Further improvement (in clinical signs and biochemistry parameters) occurred after documentation of resistance to benzylpenicillin in both the abdominal effusion and the splenic cultures, which prompted a change in antibiotic regimen. Pradofloxacin was chosen based on sensitivity testing and was preferred over other appropriate antibiotics for its bactericidal properties and good tissue penetration (Blondeau and Shebelski 2016). Its use was further justified by the possibility of addressing a potential underlying *Bartonella* spp. infection (Lappin and Fitzgerald 2024). In the present case, splenectomy alone may not have been sufficient to cure the cat, supporting the hypothesis of generalised bacteriaemia and/or sepsis.

## 3 Conclusions

Septic splenitis is an uncommon diagnosis that should be considered as a differential diagnosis in cats with splenomegaly and suspected immunosuppression and/or underlying infectious diseases. A thorough assessment for septic shock and septic peritonitis is recommended. Splenectomy should be considered in a timely manner, particularly in the absence of a clinical response to antibiotics. Antibiotic use should be based on bacterial culture and sensitivity testing.

## Author Contributions

Martina Vecín Sancho: data curation, formal analysis, investigation, visualisation, writing – original draft, writing – review and editing. Perrine Henry: conceptualisation, formal analysis, investigation, project administration, visualisation, supervision, writing – original draft, writing – review and editing. Mica Taylor: writing – original draft, writing – review and editing. Catherine Davidson: writing – review and editing. María Lázaro Muñoz: writing – review and editing. Maša Vilfan: writing – original draft, writing – review and editing. Efa Llewellyn: writing – review and editing. Paola Cazzini: writing – original draft, writing – review and editing. Linda Morrison: writing – review and editing. Kelly Blacklock: writing – review and editing. Danielle Gunn‐Moore: conceptualisation, supervision, writing – original draft, writing – review and editing.

## Funding

The authors have nothing to report.

## Ethics Statement

The authors have nothing to report.

## Patient Consent Statement

Informed consent (written) was obtained from the owner or legal custodian of this animal described in this work for all procedures undertaken and for the use of their data. No animals or people can be identified within this publication.

## Conflicts of Interest

The authors declare no conflicts of interest.

## Supporting information




**Supplementary Table 1**: Clinical parameters, medical and dietary management over the course of treatment of an Abyssinian cat diagnosed with septic splenitis.

## Data Availability

The corresponding author can provide the data that support the findings of this study upon reasonable request.
