# Supplementary material for: Successful Management of Septic Splenitis in an Abyssinian Cat
Source: Vet Med Sci. 2026 Apr 10;12(3):e70943. doi: 10.1002/vms3.70943 (PMC13067983; doi:10.1002/vms3.70943)
Supplement: Supplementary file 1 — Supplementary Table 1: Clinical parameters, medical and dietary management over the course of treatment of an Abyssinian cat diagnosed with septic splenitis. [file VMS3-12-e70943-s001.docx]

Supplementary Table 1: Clinical parameters, medical and dietary management over the course of treatment of an Abyssinian cat diagnosed with septic splenitis.

| Date | 31/12/2024 | 01/01/2025 | 02/01/2025 | 03/01/2025  splenectomy | 04/01/2025 | 05/01/2025 | 06/01/2025  Discharged at 4pm | 08/01/2025  Bacteriology results | 14/01/2025  Revisit 1  Feeding tube out | 31/01/2025  Revisit 2 |
| --- | --- | --- | --- | --- | --- | --- | --- | --- | --- | --- |
| Body weight (kg) | 2.95 | 3.02 | 3.02 | 2.97 | 3.0 | 2.99 | 3.07 |  | 2.97 | 3.09 |
| Temperature (degree C) | 38.9 to 39.9 | 38 to 38.9 | 38.5 | 38.8 | Not recorded | 38.1 | 38.1 |  | 38.3 | 37.4 |
| Pulse (bpm) | 208 | 208 | 220 | 220 | 200 | 206 | 192 |  | 180 | 204 |
| Respiratory rate (bpm) | 40 | 28 | 32 | 40 | 32 | 24 | 28 |  | 20 | 36 |
| Maropitant 1mg/kg IV SID | Yes | Yes | Yes | Yes | Yes | Yes | Yes |  | No | No |
| Amoxicillin and clavulanic acid 20mg/kg IV TID | Yes | Yes | Yes | Yes | Yes | Yes | Yes | Stopped | No | No |
| Buprenorphine 20 ug/kg IV TID | Yes | Yes | No | No | Yes | Yes | No |  | No | No |
| Enrofloxacin 5mg/kg PO SID | No | No | No | No | No | No | No | Started | Yes | Yes |
| Methadone 0.1mg/kg IV q4h | No | No | Yes | Increased to 0.2mg/kg | No | No | No |  | No | No |
| Gabapentin 50mg PO TID | Yes | No | No | Yes | Yes | Yes | No | Yes | Decreased 25mg TID | Stopped |
| IV fluid therapy | No | 2mL/kg/h | 2mL/kg/h | 2mL/kg/h | Stopped | No | No |  | No | No |
| Food intake (calculated RER = 160 kcal)   - Spontaneous - Feeding tube | 22.2 kcal | 29.6kcal | 0kcal | 20kcal  14kcal | 85kcal | 92.5kcal  80kcal | 29.6kcal  15kcal |  | 100% | 100% |
